# Glass Fibers Reinforced Concrete: Overview on Mechanical, Durability and Microstructure Analysis

**DOI:** 10.3390/ma15155111

**Published:** 2022-07-22

**Authors:** Jawad Ahmad, Roberto Alonso González-Lezcano, Ali Majdi, Nabil Ben Kahla, Ahmed Farouk Deifalla, Mohammed A. El-Shorbagy

**Affiliations:** 1 Department of Civil Engineering, Military College of Engineering, Risalpur, Sub Campus of National University of Sciences and Technology, Islamabad 44000, Pakistan; jawadcivil13@scetwah.edu.pk; 2 Architecture and Design Department, Escuela Politécnica Superior, Campus Montepríncipe, University San Pablo CEU, Alcorcón, 28925 Madrid, Spain; 3 Department of Building and Construction Technologies and Engineering, Al-Mustaqbal University College, Hillah 51001, Iraq; alimajdi@mustaqbal-college.edu.iq; 4 Department of Civil Engineering, College of Engineering, King Khalid University, Abha 61421, Saudi Arabia; nbohlal@kku.edu.sa; 5 Structural Engineering Department, Faculty of Engineering and Technology, Future University in Egypt, New Cairo 11845, Egypt; ahmed.deifalla@fue.edu.eg; 6 Department of Mathematics, College of Science and Humanities in Al-Kharj, Prince Sattam bin Abdulaziz University, Al-Kharj 11942, Saudi Arabia; ma.hassan@psau.edu.sa

**Keywords:** glass fiber, compressive strength, durability, scanning electron microscopy

## Abstract

Prior studies in the literature show promising results regarding the improvements in strength and durability of concrete upon incorporation of glass fibers into concrete formulations. However, the knowledge regarding glass fiber usage in concrete is scattered. Moreover, this makes it challenging to understand the behavior of glass fiber-reinforced concrete. Therefore, a detailed review is required on glass fiber-reinforced concrete. This paper provides a compressive analysis of glass fiber-reinforced composites. All-important properties of concrete such as flowability, compressive, flexural, tensile strength and modulus of elasticity were presented in this review article. Furthermore, durability aspects such as chloride ion penetration, water absorption, ultrasonic pulse velocity (UPV) and acid resistance were also considered. Finally, the bond strength of the fiber and cement paste was examined via scanning electron microscopy. Results indicate that glass fibers improved concrete’s strength and durability but decreased the concrete’s flowability. Higher glass fiber doses slightly decreased the mechanical performance of concrete due to lack of workability. The typical optimum dose is recommended at 2.0%. However, a higher dose of plasticizer was recommended for a higher dose of glass fiber (beyond 2.0%). The review also identifies research gaps that should be addressed in future studies.

## 1. Introduction 

The construction industry is one of the world’s most rapidly growing sectors. Concrete is a widely applied construction material in the construction industry and is critical to its success [1,2]. Concrete is malleable and flowable while wet, but once set, it is tough and long-lasting [3]. Concrete will be the most frequently used building material globally due to its diverse and favorable features. On the other hand, concrete has several undesirable features such as brittleness, poor fracture resistance, low impact resistance, and high weight. Therefore, there is a demand to enhance the concrete assets [4].

In compression, conventional concrete (CC) is usually strong but not in the domain of tension [5,6]. When tensile stresses are formed or tension zones are detected, steel bars are often used to compensate in conventional concrete, also known as reinforced concrete. Fibers are introduced into concrete to boost its essential tensile capacity, resulting in fiber-reinforced concrete, a particular kind of concrete (FRC) [7,8,9].

Over the past few decades, there has been a growing concern about applying fibers instead of conventional materials to reinforce or rehabilitate concrete structures [10,11,12,13]. FRC is described as concrete formed using hydraulic cement and fine and coarse aggregates reinforced with fibers permitting ACI [14]. Glass fiber is a well-known fiber that, owing to its minimal specific weight and water absorption, as well as its large elastic modulus, tensile strength, and weight, bears a lot of promise for concrete reinforcement [14].

Fibers may lower the overall cost of construction by substituting the conventional and energy-intensive techniques of wiring mesh and steel reinforcing bars with fibers [15]. As a result, labor costs will be lower, maintenance costs will be lower, construction time will be saved, and the build cost will be lower. Because the amount of fiber used is often much less than the volume of raw material needed to create conventional reinforcement, energy will also be saved. The spinneret process, which is analogous to the manufacturing of continuous basalt fibers [16], is used to produce glass fibers. During the manufacture of glass fibers, overhead gas burners are often utilized to heat the melt. Glass has a melting point of 1400–1600 °C. Glass fibers are less resistant to alkali than basalt fibers but perform better than basalt when exposed to acids [17]. The primary drawback of glass is that it degrades in alkaline settings due to its low alkali resistance [15]. The alkalinity of concrete restricts its uses.

Glass fibers (GF) were first employed as a mortar and concrete reinforcement in 1931 [18]. They are made by drawing molten glass through spherical holes, stranding roughly 200–240 individual fibers, and then cutting them into smaller portions [19]. Glass fiber is a waste product dumped in significant quantities by the glass producing industries. As a result, the application of such fibers may improve the mechanical capacity of concrete while also facilitating the disposal of industrial waste [20]. Polyester resin is reinforced with glass fiber in glass fiber-reinforced plastic (GRP) composites. Construction, aerospace, automotive, and locomotive sectors produce waste, which is a discarded byproduct of production and is removed at the end of its service life. GRP waste is created in the United Kingdom (UK) at a rate of roughly 55,000 tons per year, with the amount predicted to rise by 10% every year [21]. The physical properties of glass fibers are presented in Table 1. Glass comes in a wide range of colors, chemical compositions, and properties [22]. Glass fibers show excellent strength [23,24], thermal characteristics, durability and interfacial attachment to the matrix [25]. Glass fibers are often utilized as reinforcement in resins and composites because of their incredible strength qualities [26].

**Figure 1:**
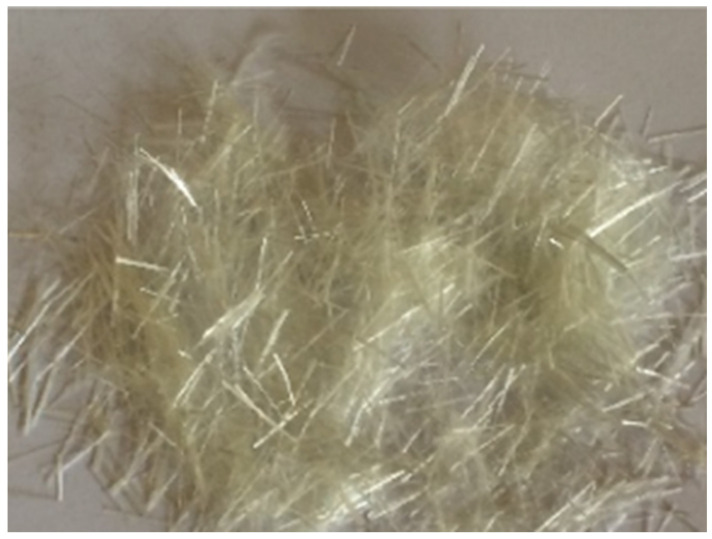
Glass fiber: used with permission from Elsevier [24].

The color of glass fiber used in concrete is mostly light white, as shown in Figure 1. The typical glass fiber varies from 6 to 18mm with an average density of 2.60 g/cm^3^. The tensile strength of glass fibers ranges from 1000 MPa to 3400 MPa with elastic modulus from 29 to 84 GPa. Furthermore, GF has a significantly higher modulus of elasticity than synthetic fibers, although not as high as steel or carbon fibers. They can withstand high temperatures and begin to weaken at about 700–900 °C [27]. The high sensitivity to water and low alkali resistance in alkali conditions are disadvantages of GF [28]. This is because they wash away alkali metal ions from their exterior surfaces, causing cracks [27]. Therefore, throughout the silane preparation process, GF is shielded from the detrimental effects of moisture in the atmosphere [29].

Various studies have also shown that fiber incorporation in the concrete improves engineering assets of concrete such as tensile capacity, flexural capacity, impact resistance, ductility and force carrying capacity even after cracks [34]. Nevertheless, the impact of fiber incorporation on concrete compressive capacity is still debatable, as some researchers found that it enhanced compressive ability while others found that it reduced it [35,36]. Some researchers [37,38] even concluded that fiber addition had no influence on compressive capacity. GF inclusion in concrete mixes may manage the concrete’s durability against shrinkage, according to studies on GF-reinforced concrete [39], increase their compressive and flexural capacity [40,41] and increase their hardness and other properties related to it [42,43]. Fibers are frequently scattered out across the concrete during mixing. This enhances the mechanical qualities and boosts the interlocking strength [44].

The fiber type, length, diameter, and quantity of fiber employed may all influence the implementation of fiber-reinforced composites. High fiber content is generally required for composites to work well and achieve high performance. For improved concrete performance, the ideal content is equally vital. Many researchers are focusing on glass fibers rather than steel fibers. Steel fibers are expensive and prone to thermal expansion and corrosion problems. Nevertheless, the understanding of glass fibers in concrete is sporadic, and no one can simply assess its significance in concrete. As a result, this study emphasizes the physical qualities of glass fibers, as well as the fresh assets, mechanical properties, and durability of concrete with the incorporation of glass fibers. A good outcome will also inspire future researchers to examine the selection and employment of glass fibers in practical applications.

## 2. Fresh Properties

### Slump Flow 

**Figure 2:**
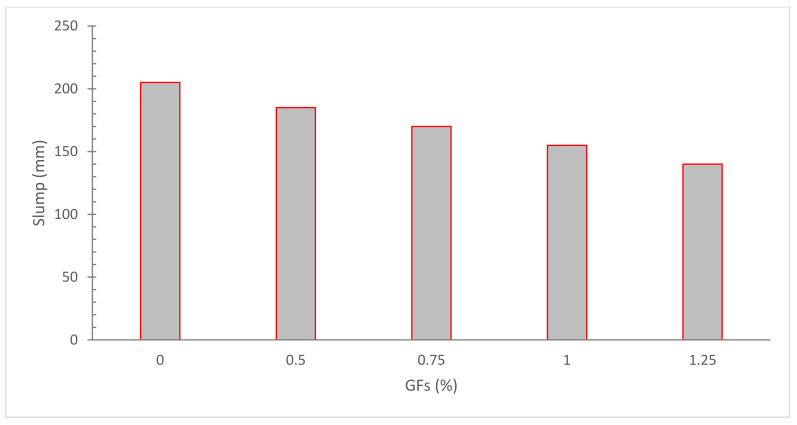
Slump Flow: Data Source [45].

Figure 2 shows the slump flow of concrete with the incorporation of glass fibers. Clearly, the fluidity of concrete decreased with the inclusion of glass fibers.

Song et al. [46] discovered that the flowability of short length fibers were lower than larger length fibers. Limited-length fibers provide a larger surface area for enhancing the binding of fiber cement paste [47]. Because glass fibers have a longer length than nylon fibers, they have a shorter active surface area for forming fiber paste bonds when associated to nylon fiber. Glass fiber-reinforced concrete had a slightly larger slump flow than nylon fiber-reinforced concrete. Furthermore, since glass fibers are less ductile (stiffer), they provide greater resistance to concrete flow, resulting in less workable concrete [22]. The glass fibers produced considerably greater slump values than the nylon fibers. At a scope of 5–10 mm, slump values increased, with the maximum variation between glass and nylon fibers at 6% of fibers, and the value of slump of glass fibers is 9% greater than nylon fibers. Workability decreased as the fiber dose rose, with 1.5 percent fiber prescription having the lowest flow value. Glass fibers have a negligible impact on the compressive capacity of glass fiber-reinforced concrete. The use of glass fibers boosted the final, binding strength of concrete [48]. The slump test findings show that when GF dose and metakaolin concentrations rise, the spread diameter decreases [49]. Because fibers have a larger surface area, they require additional water to cover them, decreasing the amount of water available for greasing. Furthermore, the presence of fibers increased internal friction between concrete pieces, requiring the use of additional cement paste [38]. The slump was decreased to 50 mm after adding 0.25 percent fiber. With increasing fiber content, the slumps of the following versions imply declining values. The surface form and physical characteristics of coir fibers contribute to this proclivity [50]. Fibers have hydrophilic surfaces, according to one study, and hence resist water [5]. In a similar line, multiple findings have found that adding fibers reduces the slump value [51,52,53]. 

## 3. Mechanical Properties 

### 3.1. Compressive Strength (CPM)

**Figure 3:**
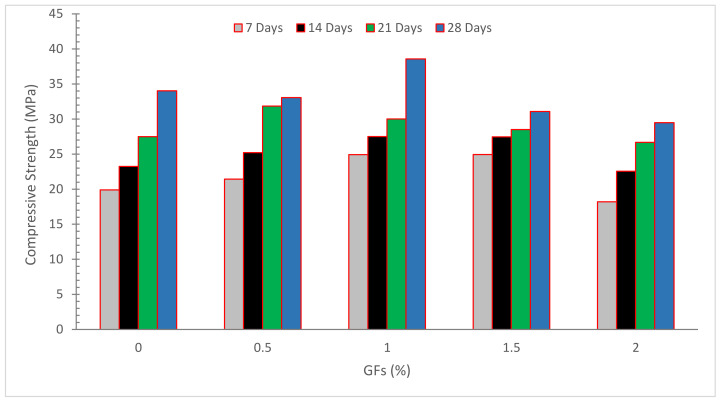
Compressive strength: Data source [54].

Figure 3 demonstrates the compressive strength (CPM) of concrete with the inclusion of glass fibers. The CPM of concrete improved to a certain extent with the incorporation of glass fibers.

Our study indicated that age bears no effect on the CPM of glass fiber-reinforced concrete. GF failed to enhance the CPM of the sample as compared to control specimens. In the presence of GF, it is impossible to achieve consistent distribution of concrete ingredients. These results corroborate those of Fenu et al. [55]. When glass fibers are introduced to reinforced concrete composites, they act as a connecting agent. Distributing stress from glass fibers to the substrate offers a resistance of samples to crack initiation in terms of interface shear resistance. The CPM of samples containing 0.25, 0.75, and 1.25 percent GF rose by 25.52, 39.57 and 47.92 percent, correspondingly, when self-compacting concrete was used. Glass fibers may be preventing fractures in specimens under CPM, which explains the enhancement [56]. Nylon and glass fiber expansion was reduced due to the holding of the fiber cement paste interface and improved concrete CPM [46]. Except in certain pozzolan-containing mix designs, no increase in CPM was seen when the glass fiber concentration was increased from 1.5 percent to 2.5 percent. In this study, it was discovered that adding pozzolanic admixtures to GFRC specimens resulted in a modest reduction in CPM in the short term. The specimens containing nano silica had the lowest value, most likely owing to the severe decrease in workability caused by the combined presence of nano silica and glass fibers. It can be inferred that by changing the kind and amount of pozzolanic materials used, GFRC specimens’ CPM increased significantly over time. This increase in strength is due to the high amount of silica in pozzolanic materials, which boosts concrete strength by creating calcium–silicate–hydrate (C–S–H) gel during the hydration process. When comparing the improvements made, it was discovered that the mix containing 15% metakaolin exhibited the maximum CPM when associated with the other mix types. The addition of glass fibers to concrete does not result in better CPM than PC. Furthermore, the presence of pozzolanic components in GFRC specimens reduces CPM in the near term. With time, CPM improves. In fact, the cement matrix has a greater impact on glass fiber concrete CPM than aging glass fiber degradation. The inclusion of metakaolin resulted in the greatest improvement in CPM [33]. This owes to the fact that the pozzolanic reaction is slower than cement hydration. Glass fibers with a 0.5 percent replacement showed slightly better CPM than control concrete, whereas glass fibers with 1.0 and 1.5 percent replacement had approximately identical CPM to control concrete. This means that adding glass fibers has a negligible impact on CPM [48]. 

### 3.2. Flexural Strength (FLS)

**Figure 4:**
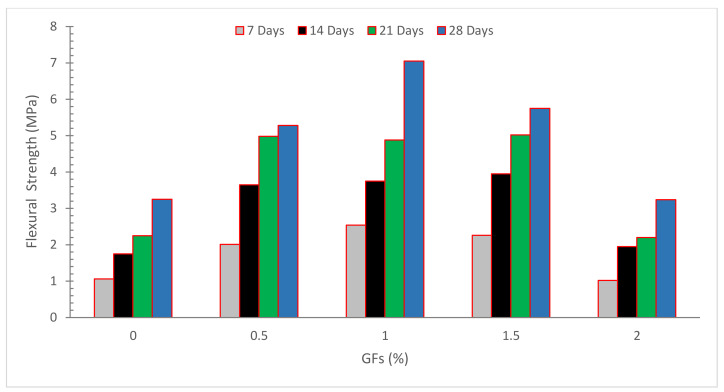
Flexural strength: Data source [54].

Figure 4 shows the flexural strength (FLS) of concrete with the inclusion of glass fibers. It can be seen that concrete FLS of concrete improved up to a certain extent with the incorporation of glass fibers in a similar manner to CPM. The inclusion of basalt and glass fibers increased the FLS of the material. The improvement in strength was more noticeable in the case of basalt fiber, which showed a steady rise with increasing fiber dose. However, following a 0.50 percent dose of glass fiber, no significant change in FLS was found [24]. Increasing the amount of glass fiber in a beam improves its flexural performance and FLS. Moreover, increasing the quantity of recycled glass fiber enhances the distribution of FLS results, similar to compressive strength, leading to a maximum coefficient of variance (COV) of 9.61 percent for a sample reinforced with 1.25 percent of glass fiber [56]. The bridging effect of GFRP fibers, by which the bridging fibers may partly transmit the stress across the crack, may improve the flexural performance of reinforced beams using recycled GFRP fibers [56]. When 0.1 percent glass fibers are added to conventional concrete, FLS increases by 19.31 percent after 28 days [57]. The FLS of the specimens reinforced by carbon-fiber grid and steel-strand grid increased by 47.7% and 13.3%, respectively, as compared to the normal specimen [58]. When the FLS of concrete containing 1% polyethylene terephthalate is compared to the FLS of control concrete, the FLS of the control concrete improves by 20% [59]. Bis-acryl composite resin had the greatest FLS in both glass and polyethylene fiber reinforcement, followed by heat-cure and self-cure resin [60]. Glass fiber significantly enhanced the FLS of concrete, according to research [61]. Compared to control cement-based mortar samples, the findings obtained with varied nylon percentages showed an improvement in FLS up to 13% [62]. Polyethylene fibers did not boost the FLS of hybrid composites, according to research [63]. Glass fibers exhibit a brittle failure type. However, polyethylene fibers prevent shattered specimens from completely separating [23,61]. Nylon fibers boosted FLS more efficiently than glass fibers, according to research [25]. The FLS rose as the GF volume percentage increased. This most likely owes to the fact that when the matrix cracks, the fibers will resist the load until the interfacial link between the fibers and the matrix breaks down [31]. 

**Figure 5:**
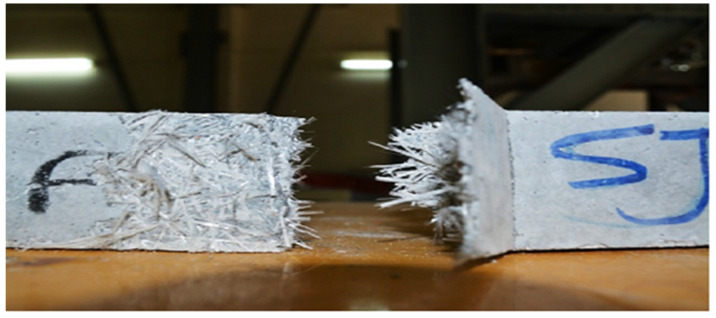
Failure of GF-reinforced beam: Permission of use from Elsevier [33].

In the glass fiber specimens, point accumulation of glass fibers and a lack of a proper mortar cover surrounding the fibers cause cracks to appear around the fibers, eventually leading to concrete rupture as shown in Figure 5. Some studies reported that fibers (steel or nylon) improved flexural strength due to crack prevention. However, glass fibers are also brittle, resulting in brittle failure with any warning (deformation). As a result, supplementary cementitious elements are recommended as a way to increase the flexural capacity of glass fiber-reinforced concrete [5,38]. 

**Figure 6:**
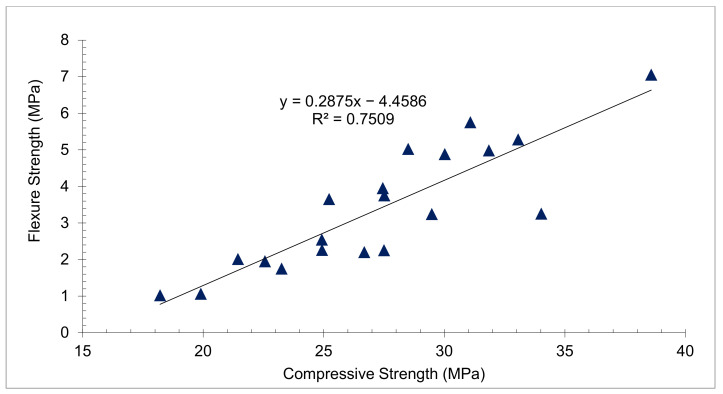
Linear Regression between Compressive and Flexural Strength: Data Source [54].

The relationship between CPM and FLS is seen in Figure 6. With an R square of 0.75, the regression line seems to be straight, indicating a significant relationship between compressive and flexural strength. As a result, the equation indicated in Figure 6 may be used to estimate FLS from concrete CPS.

### 3.3. Split Tensile Strength

**Figure 7:**
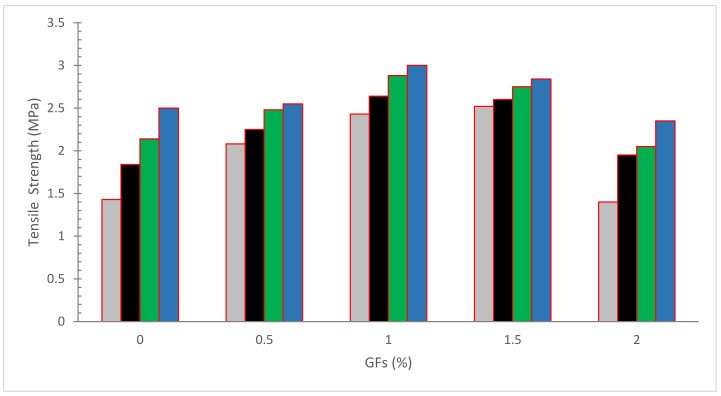
Tensile strength: Data source [54].

Figure 7 shows the tensile strength (TS) of concrete with the inclusion of glass fibers. It can be seen that concrete TS of concrete improved up to a certain extent with the incorporation of glass fibers in a similar way to CPM. The addition of GF to concrete and mortar enhanced the splitting tensile strength by up to 12.5 percent and 17 percent, respectively, according to Muna and Jallo [64]. The rise was attributable to the fibers’ bridging activity across the fractures, which initially limited the growth of microcracks. After flexural failure, the stress is transmitted to the bridging fibers, which slows the crack development and increases the splitting tensile strength. However, after a 0.50 percent dose of GF, no significant improvement in strength was seen [24]. The optimal glass fiber inclusion level is 1.5 percent, with 14.29, 8.12, and 10.03 percent increases in compressive, flexural, and split tensile strengths, respectively [65]. Macro poly fibers are a kind of macro synthetic polypropylene fiber (PP) created and produced in a new continuously deformed shape for excellent performance and optimal anchoring to concrete, making them more structurally compliant [66]. The drying shrinkage of multi-filament and fibrillated fibrous concrete was reduced by 40% compared to standard concrete; this decrease is due to the greater tensile strength of synthetic fibers, which helps bear additional loads. Compared to concrete without fibers, synthetic fibers enhanced residual tensile strength and consequently improved ductility [67]. Microfibers prevent microcracks from forming at the start of the concrete hardening process and boost tensile strength. Microfibers also serve as a load-bearing component, preventing macrocracks and increasing concrete ductility [68]. The ultimate tensile strength of 1.5 percent polypropylene microfiber was 21.4 percent greater than ordinary concrete [69]. When 0.1 percent glass fiber is added to conventional concrete, the splitting tensile strength increases by 42.23 percent after 28 days [57]. When compared to control cement-based mortar samples, the findings obtained with varied nylon percentages showed an improvement in tensile strength (up to 21%) [62]. The composite specimens perform well in the axial track than in the oblique track and in terms of fiber composition, composites containing 20% sisal fiber and 80% GF perform superior for tensile loading while pure GF concrete performs better for flexural loading. The pure glass fiber-reinforced sample has a maximum tensile strength of 181.84 MPa and a highest flexural capacity of 288.80 MPa [30]. The findings reveal that adding GF fibers to the mix has no influence on compressive strength. However, when the percentage of glass fibers was increased, the tensile capacity and flexural capacity of GF rose, which might be attributable to the bridging action of concrete [70]. Table 2 shows the summary of mechanical strength of concrete with the inclusion of GF.

**Figure 8:**
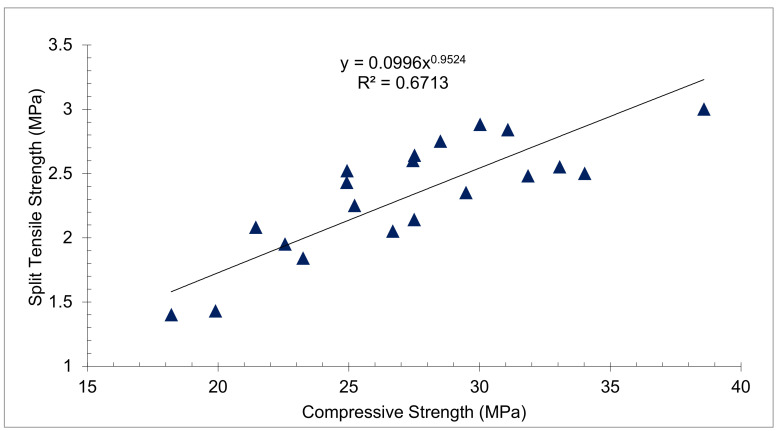
Linear Regression between Compressive and Tensile Strength: Data Source [54].

Figure 8 shows the relationship between CPM and TS. The regression line appears straight with an R square of 0.67 which shows a strong correlation between CPM and TS. As a result, the equation presented in Figure 8 may be applied to forecast the tensile strength of concrete based on its compressive strength.

### 3.4. Elastic Modulus 

**Figure 9:**
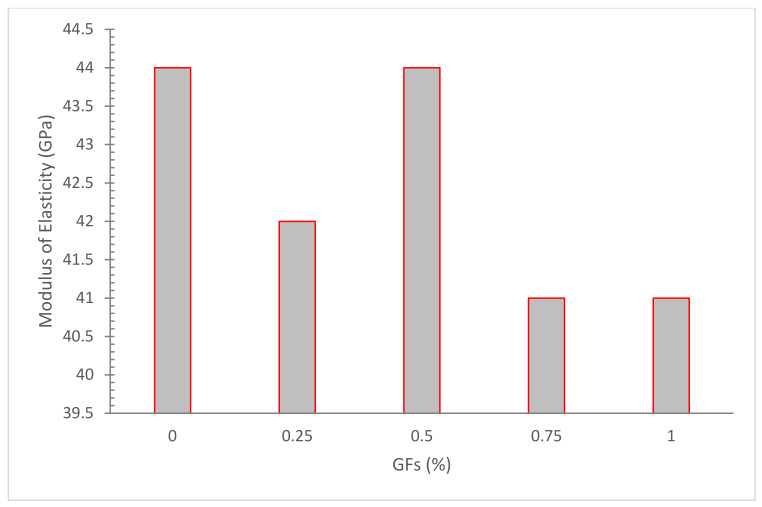
Elastic Modulus: Data Source [24].

Figure 9 depicts the modulus of elasticity of glass fibers at various fiber volume fractions. The modulus of elasticity in fiber-reinforced concrete decreased somewhat with increased fiber dose when compared to ordinary concrete. 

According to one study, increasing the volume percentage of glass fibers in self-compacting concrete (SCC) from 0 to 1.5 percent enhanced the modulus of elasticity [31]. A study reported that 0.25 percent glass fibers resulted in a 7 percent drop in modulus of elasticity as compared to control concrete. Figure 9 further shows that the modulus of elasticity of concrete containing glass fibers was somewhat lower than that of banana fibers. This is due to the fact that glass fibers have a lower modulus of elasticity than banana fibers [24]. The research also discovered that SCC with fibers had a roughly 10% higher modulus of elasticity than simple SCC [77]. The findings of the laboratory tests show that the use of fibers bear no significant impact on the modulus of elasticity of concrete. At low glass fiber concentrations, there was primarily a small drop in the concrete’s modulus of elasticity [78].The inclusion of glass fibers results in minor improvements in elastic modulus of 0.5–5%, and the difference between elastic modulus values of plain concrete and 1% GF concrete is small [74]. Overall, fewer studies are available on the elastic modulus of concrete and more detailed research is required.

## 4. Durability 

### 4.1. Chloride Penetration (16)

**Figure 10:**
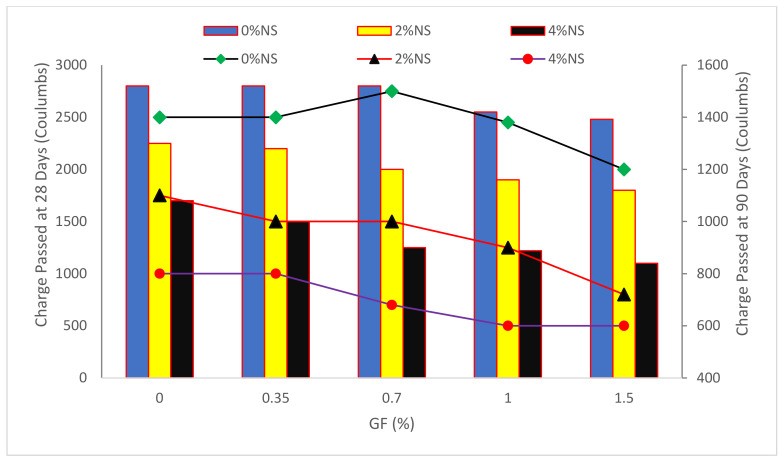
Chloride-Ion Penetration: Data Source [31].

Chlorides may cause corrosion in reinforced steel if they manage to permeate into the concrete. These represent some of the most significant problems that may be identified in concrete structures globally. Figure 10 shows the chloride-ion permeability test results as a function of nano-silica and glass fiber concentration and test ages. 

**Figure 11:**
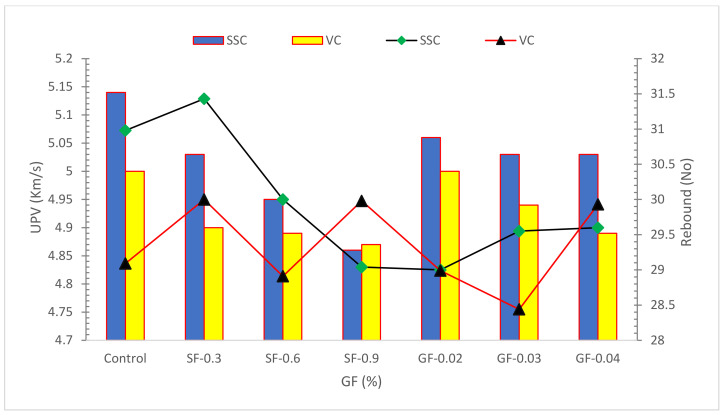
UPV and Rebound Hammer Test Results: Data Source [76].

The chloride-ion permeabilities of the SCC composites are shown in Figure 11. With increasing GF volumetric percentages and NS content, there was a gradual reduction in chloride-ion penetrations. Chloride-ion penetrations decreased between 3068 and 3004 C, and between 2678 and 2578 C, corresponding to the 28- and 90-day age periods, in mixes without NS, with GF volumetric fractions rising from 0 percent to 1.5 percent. These findings show that increasing GF and NS content has a favorable impact on chloride-ion penetrability. Based on ASTM C 1202 [79], Concrete that has been charged between 100 and 1000 degrees Celsius is thought to be resistant to chloride-ion penetration. As a result, all GF-reinforced SCC mixtures have an extremely small chloride-ion penetrability. The interaction of glass fibers with concrete increases the pore and internal structure of concrete compatibility by preventing cracks, thus increasing its density. The water and chloride penetration into the concrete is reduced due to the concrete’s more compact structure. Because glass fibers bridge across fractures and form interconnected voids, glass fiber-reinforced SCC samples have somewhat higher chloride penetrability than the reference mix sample. This was in agreement with the findings of other studies [80]. Because of the excellent connection of GF with binders, the GF-reinforced SCC has decreased chloride-ion permeability up to 0.6 percent of GF inclusion [70].

### 4.2. Water Absorption

The water absorption test determines how quickly the exterior- and inner-concrete surfaces absorb water. The test involves determining the rise in density of samples due to water absorption with time after the sample has been subjected to water. Because water includes numerous harmful substances that soak into concrete, causing concrete disintegration and resulting in decreased durability, more water absorption leads to lower durability.

The impact of GF on water absorption was much better than that of PPF. When the water/binder ratio was 0.30, adding 1.35 percent GF reduced the specimen’s water absorption from 3.49 percent to 1.99 percent, with a maximum reduction of 43.0 percent. Meanwhile, adding 0.45 percent PPF reduced water absorption from 3.49 percent to 3.12 percent, with a maximum reduction of 10.6 percent [71]. Compared to control SCC, the water absorption data demonstrate that increasing the GF concentration slightly enhances the water absorption. However, all GF-reinforced SCC had water absorption values within 2% of each other. As a result of the GF’s strong connection with cement particles, water penetration into concrete is reduced due to a more compact pore structure. The use of GF increases the concrete’s durability [70]. When steel fibers were introduced at a rate of 2.0 percent, the lowest quantity of water absorption was observed [38]. Because the elastic modulus of traditional concrete is less than that of fiber-reinforced concrete, the addition of fibers to concrete would boost the tensile strain characteristics, limiting the growth and expansion of early fissures [81]. To reiterate, increasing the density of concrete lowers water absorption. Greater doses (over 2.0 percent) resulted in the least compact concrete due to a lack of flowability. Based on one research paper, higher water absorption was seen in coconut fiber concrete than in control mortar due to the increased porosity of the coconut coir fraction mortar compared to the reference mix. The most significant factors affecting water absorption are the porous nature of the cement blocks and the existence of an interfacial zone around the particles. The results show that in comparison to a control mix, the coconut fiber mortar demonstrated much more water absorption [82].

### 4.3. Ultrasonic Pulse Velocity and Rebound Hammer Test

Ultrasonic pulse velocity (UPV) assessment validates the integrity and quality of mechanical strength of concrete by measuring the velocity and attenuation of an ultrasonic wave travelling through concrete at a depth of approximately 6 feet. Smaller-speed regions have smaller compactness and mechanical capacity than greater-speed regions. The Rebound hammer test is a non-damaging concrete assessment procedure that evaluates the concrete’s mechanical capacity rapidly and simply. The homogeneity of plain concrete may be reduced by adding fibers. Concrete is graded according to the UPV values and rebound numbers specified in the appropriate standard codes. The steel fiber-reinforced concrete surface hardness was found as a hard layer. The surface hardness of the GF-reinforced concrete matrix was lower than that of RCC, and it was evaluated as a good layer. In comparison to ordinary concrete, reinforced concrete has poor homogeneity. The reinforced concrete containing a large fiber volume improved the homogeneity in this study. As the amount of fibers rose, the pulse velocity dropped. Concrete with steel fibers have a lower pulse velocity than concrete with glass fibers. When compared to reinforced vibrated concrete, reinforced SCC exhibited higher uniformity [76].

### 4.4. Creep

Concrete creep is the deformation of a structure caused by a sustained load. When concrete is exposed to continuous pressure or stress, its shape may be altered. Over a period of 50 years, a study on the creep behavior of pultruded GFRP revealed a typical reduction in instantaneous longitudinal; flexural stiffness of pultruded profile of around 50%, and it was predicted that for a period of one year, the flexural stiffness will be in the range of 75–80% of elastic. It is also expected that after one year of loading, creep deflection would grow by up to 35 percent, and after 50 years, increase by up to 100 percent [83]. Another investigation of creep behavior on GFRP-reinforced concrete beams found that the creep effect was caused by loading and that the environment exposure was considerable, particularly at the concrete strain. The varied rheological qualities of a segment might lead to stress migration across the matrixes and towards the fibers, according to a GFRP creep research paper. The axial stresses of composites were supposed to be constant over time, while matrix stress was zero. This was most likely owing to the fiber’s lack of viscosity. Furthermore, research of GFRP creep behavior on a concrete footbridge revealed that the creep deformations of GFRP structures were reduced. The load level and ambient factors, such as temperature and humidity, have an impact on creep behavior. Strains in concrete and GFRP bars, as well as midspan deflections, were measured for all relevant environmental variables over the 10-month exposure period [84]. Research, on the other hand, reveals the findings of a creep test program on GFRP laminates and their component phases (matrix and fiber), which were subjected to various stress levels under continuous ambient circumstances. To analyze the creep behavior of a pultruded GFRP structure, it was held under load for more than two years. Due to the directional arrangement of fibers, the result showed that rheological properties of pultruded composites connected with shear loadings were independent, as shear creep was found to be larger than creep under traction, and the creep deflection was predicted to be 24.4 percent after 50 years [85]. The creep behavior of GFRP was tested, and after 6 and 14 months, Young’s modulus composites were lowered by 20%. Meanwhile, creep behavior of GFRP pultruded flexural member revealed shear stiffness after 50 years with average decrease of effective instantaneous flexural and larger due to shear deformation rather than bending counterpart. Finally, after five months, a creep analysis on GFRP girders revealed an increase in instantaneous deflection of up to 40%, proving the potential of GFRP in structural industries [86].

### 4.5. Shrinkage 

**Figure 12:**
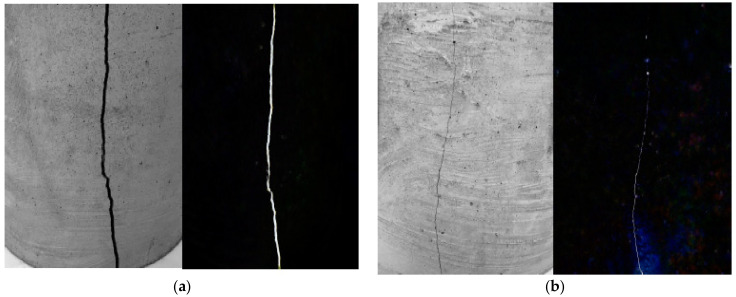
Shrinkage Crack Width (**a**) Without Fibers and (**b**) With Fibers [87].

Concrete shrinkage and volume reduction occur due to moisture loss, which eventually results in cracks and more concrete deformation. Figure 12 depicts a close-up of a fracture formed on a concrete ring surface of fiber-containing and non-fibrous specimens, demonstrating that fibers significantly decreased shrinkage. 

Early-stage shrinkage (polyolefin or capillary shrinkage), autogenous shrinkage, carbonation shrinkage, and drying shrinkage are the four types of shrinkage. Early-stage shrinkage in new concrete is caused by a moisture exchange from the surface to the environment (by evaporation) and a mass exchange via the concrete to the surface. After casting, polyolefin shrinkage is detected in the early hours, which may be avoided by optimizing the mix design and curing properly. Carbonation shrinkage is caused by interactions between hardened cement paste and carbon dioxide [88]. This process causes a delayed volume to drop in the surrounding region, making it insignificant in comparison to drying shrinkage. Humidity exchange happens because of variations in relative humidity between the environment and the concrete during drying. The bulk of volume changes due to shrinkage in high- and middle-strength concretes fit into this category. As a result, this form of shrinkage is explored in depth in this study [89]. Messan et al. [90] also looked at the effect of GF on mortar shrinkage. They discovered a considerable decrease in free early age shrinkage, which they attributed to strong GF-cementitious matrix bonding. Furthermore, the GFRC shrinkage was more homogeneous than that of mortar without fibers. Finally, the favorable impact of GF in restraining cracks and reducing the probability of their formation was established. In terms of restricted shrinkage, it was reported in [91] that 1% GF reduced constrained strains in mortar by 24% after 24 h. Furthermore, GF outperformed synthetic and metallic fibers in terms of reducing constrained shrinking. Malathy et al. [92] investigated how different GF dosages affected SF-restrained plastic shrinkage. They claimed that they could prevent cracking even at low levels of GF, such as 0.3 percent. Furthermore, the paper reported that when SF was employed, the addition of GF was essential to avoid shrinkage fractures.

### 4.6. Performance of Glass Fiber under NaCl Solution 

**Figure 13:**
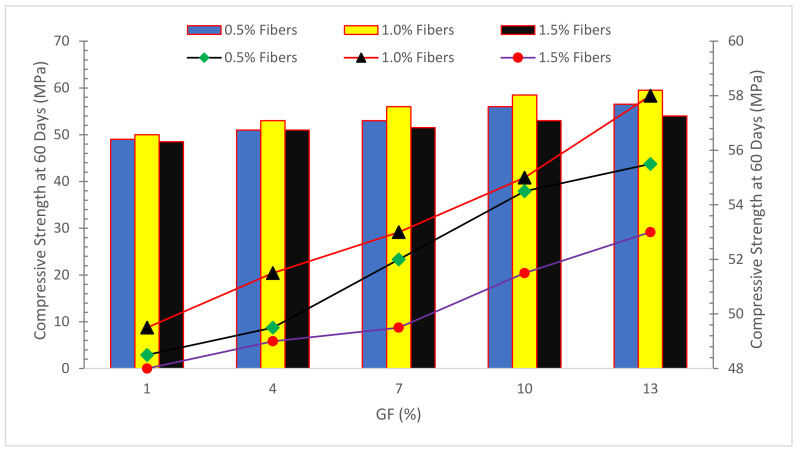
Compressive Strength Under NaCl Solution With Different PH Value: Data Source [48].

Figure 13 depicts the compressive capacity of glass fiber concrete after 60 and 90 days of salt solution exposure at various PH values. Specimens with a 1 percent fiber dose produced the highest strengths for the respective PH levels and exposure times, followed by specimens with a 0.5 percent fiber dosage. The compressive strength of each dose of concrete specimen decreased as the PH of the salt solution decreased from PH 13 to PH 1. This owes to the corrosive effects of a high acid concentration in a low PH environment. The degradation of concrete was significantly more severe owing to the entrance of Cl and H+ ions into the concrete when the aggressive character of the salt solution was enhanced by decreasing the PH from 4 to 1. Due to the acid assault, the H+ ions from sulphuric acid damage the concrete, and diffusion of the Cl ions increases in speed, resulting in a considerable reduction in compressive strength [93]. Because of its alkalinity, salt solutions with PH 10 and 13 had little effect, and concrete degradation was extremely minor, resulting in greater compressive capacity retention values. The preservation of compressive capacity decreases as the aggressiveness of the exposure rises, which may be linked to the severe degradation of concrete composites. The 1.0 percent glass fiber dose was proved to retain compressive strength better than the 0.5 and 1.5 percent fiber dosages [48].

## 5. Scanning Electron Microscopy 

**Figure 14:**
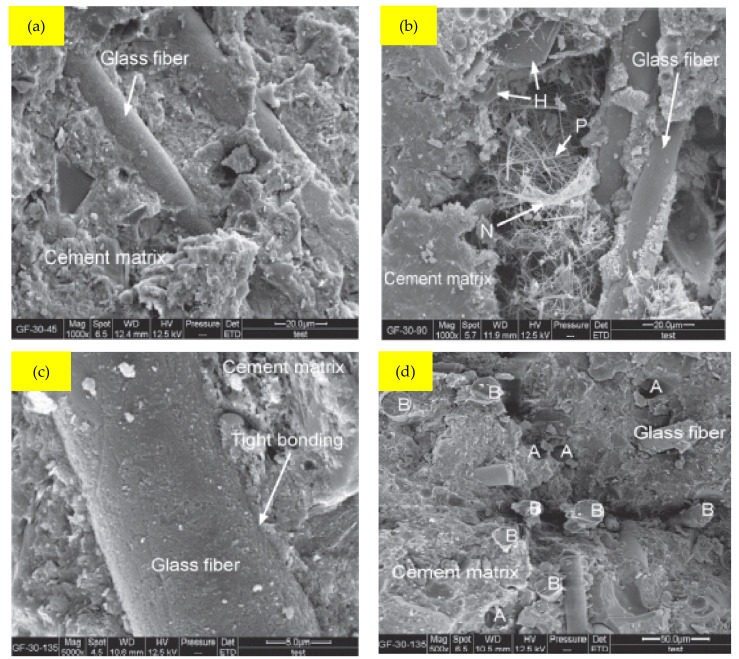
SEM Results of GF at Water to Binder Ratio 0.30: Used with the Permission of Elsevier [71].

When w/b is 0.30, SEM pictures of GFRC specimens are shown in Figure 14. The performance of RRC, as a multi-element, multi-phase, and multi-interface composite material, is substantially determined by the characteristics of the fiber–cement interface. The GF and cement matrix were extremely closely linked, as shown in Figure 14a and c, which may be explained by the fact that GF is a mineral fiber material with excellent hydrophilicity [94]. As shown in Figure 14b, it was possible to clearly see hexagonal crystalline products, needle-shaped prismatic crystals, and a reticular network crystalline structure in the pores of the cement matrix. The porosity was decreased by the GF, which filled the holes on its surface and was coated in a firmly solidified cement matrix.

The excellent fiber–matrix bonding quality made it possible for GF to increase the concrete’s strength qualities and reduce water absorption. The GF filled the gaps and decreased porosity since its surface was covered by a firmly cemented cement matrix. The GF-30-135 45 cement matrix structure (water to binder ratio 0.30 and GF 1.35 percent) was dense and less porous, as illustrated in Figure 14d, and the GF created a three-dimensional staggered support network within the matrix. The existence of the GF prevented the extension of the crack tip when fractures occurred, due to the lesser thickness of the fiber and the small space among GF. Hence, the break might only remain to spread by bypassing the GF, breaking down the GF, or dragging the fiber out. The growth of the fissures, fiber pullout, and fiber fracture, when combined with the strong GF-based concrete binding characteristic, require enormous quantities of energy. As a result, GF-30-compressive, 135’s flexural, and splitting tensile strengths were considerably enhanced. H denotes hexagonal crystalline products; P denotes needle-shaped prismatic crystals; N denotes reticular network crystalline structure; A denotes GF pullout; B denotes GF breakage.

**Figure 15:**
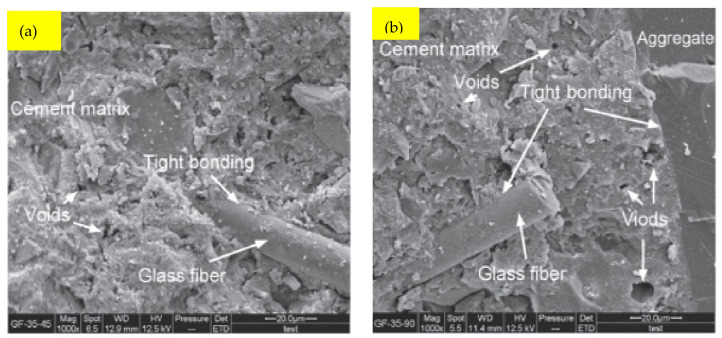

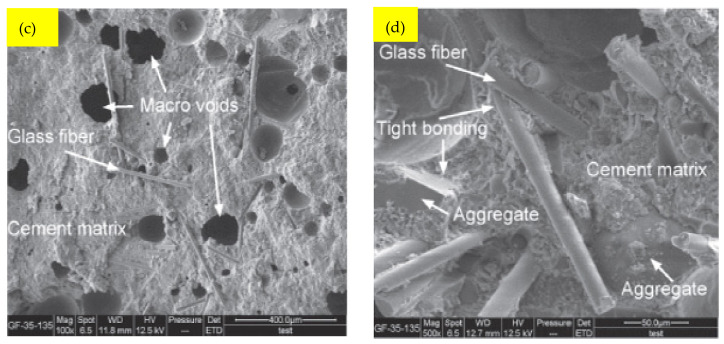
SEM Results of GF at Water to Binder Ratio 0.35: Used with the Permission of Elsevier [71].

When w/b is 0.35, the SEM images of the GFRC sample are shown in Figure 15. Even when the GF dose reached 1.35 percent, the binding condition among the GF and paste, as well as between the aggregate and fibers, was outstanding, as indicated in Figure 15a,b,d. However, as seen in Figure 15a–c, the holes and voids in the matrix grew in both size and number when the GF dose was raised. The GF-35-45 cement matrix (water to binder ratio 0.35 and GF 0.45 percent) contained a significant number of holes and voids ranging in diameter from 1 to 5 microns, and the pore size was rather consistent. Voids with a diameter of about 10 microns developed in GF-35-90 (water to binder ratio 0.35 and GF 0.90 percent). However, their quantity was quite minor. However, when the GF dose was raised to 1.35 percent, a substantial amount of macro cavities appeared, with sizes reaching or surpassing 100 microns. These macro voids were the primary cause of GF-35-rapid 135’s loss of strength and continuous increase in water absorption (water to binder ratio 0.35 and GF 1.35 percent), and when the water/binder ratio was enhanced to 0.35, the ideal GF content for compressive capacity and flexural capacity dropped from 1.35 percent to 0.90 percent. The purpose of the SEM experiment was to further investigate the connection between GF and mortar. Glass fiber dosages of 1 and 1.5 percent are shown in these photos. It was discovered that the connection between concrete and fibers was satisfactory, resulting in higher reinforcing steel and concrete bond strength [48]. Furthermore, proper packing may have contributed to the lack of fractures when shrinkage and void size reduction occurred. GF filled gaps and reduced concrete permeability, according to the study described in [95]. It was also discovered that a hybrid combination including both GF and PPF produced the greatest results in terms of void size. PPF with a lower diameter might link smaller voids, while GF with a larger diameter could connect larger ones. To summarize, the inclusion of GF results in a material that is harder to break, denser, and more impermeable. This result is also in line with the findings of Chen et al. in [96]. They also discovered that SF improved the performance of glass fiber-reinforced mortars by protecting the fibers from ageing caused by alkali environment degradation. Specifically, SF provided a barrier between the fiber and the alkali environment by covering the GF surface. Some studies using SEM revealed that GF has a propensity to form bundles and flocculate, resulting in nonuniform fiber distribution and decrease of bonding strength [97]. Therefore, care must be used while mixing concrete and selecting the fiber dose.

## 6. Factors to Be Considered while Using Glass Fibers 

It has been stressed in numerous research works and studies that the content of the fibers used to strengthen the composite material is quite significant. A greater fiber content is not always beneficial. Although fiber reinforcement is known to increase material mechanical characteristics, it generally only does so to a certain amount, beyond which the qualities begin to deteriorate. Therefore, a composite material’s ideal fiber composition is critical for achieving the greatest outcomes. Physical parameters such as fiber length, fiber count, and fiber distribution should be considered [98]. In concrete, as the amount of fibers grow, the workability of the mixture declines, necessitating the use of additives [99]. Water repellency, alkaline treatment, and surface enhancement are all examples of treatments that may be utilized to improve the qualities of fibers [100].

Other matrices have been employed to generate high-performance composites using fibers [101]. The mechanical behavior of the composite is influenced by the physicochemical interaction between the components of the composite, the degree of adhesion, and the bonding between the fibers and the matrix [102]. A robust contact between the fibers and the matrix is usually responsible for the best composite characteristics [103]. Coupling agents and other additives have been utilized to strengthen the adhesion between the composites, resulting in stronger linkages. They aid in the bonding and uniting of the fibers with the matrix [103], resulting in a composite with superior characteristics.

## 7. Application Glass Fibers

GFRC is becoming an increasingly popular and commonly utilized material. Both in structural materials, and items associated with the construction process. In addition, modest architectural features, are being manufactured with GF to create more appealing, durable, and safer public places [104]. Glass fiber-reinforced polypropylene composites are becoming more popular in the automobile sector due to their good mechanical qualities, ease of fabrication, lightweight, and low cost [101]. These composites have been used to make products, including bus bumpers and automobile seats [101]. Glass fibers offer promise as well, although their resistance to alkali conditions is poor [15]. Khan and Ali [105] examined the use of glass and nylon fibers in concrete bridge decks to reduce early age micro cracking. When compared to the control sample, the inclusion of the fibers resulted in a decrease in compressive strength and before cracking energy absorption, but an increase in toughness and flexural and splitting tensile stress [105]. Overall, the addition of nylon and glass fibers to concrete bridge decks was shown to be effective in preventing early age micro cracking [105]. Prefabricated façade panels for residential and cultural buildings, airports, stadiums, museums, and galleries are produced from GFRC. They are stronger than standard reinforced-concrete facades, and also have better resistance to temperature fluctuations, solar radiation, pollution, and corrosion, as well as hydrophobic properties [106]. Glass fiber-reinforced concrete is lighter in weight and higher in tensile strength compared to concrete. This prompted a recent research effort to investigate its feasibility as a structural material [107]. The study was conducted in collaboration with concrete precast enterprises, for whom the enhanced properties are particularly desirable since the precast pieces’ decreased weight is critical for shipping and installation. Reinforcement methods, such as carbon or glass strands and stainless steel bars, were also investigated in order to achieve a GRC with high endurance, resulting in corrosion-free solutions [108].

One of the most troubling issues in the early stages of GRC development was the durability of the glass fibers, which grew brittle over time owing to the alkalinity of the cement mortar. Significant progress has been achieved since then, and the issue is now essentially eliminated with new forms of alkali-resistant glass fibers and mortar additives that inhibit the processes behind GRC embrittlement [106].

## 8. Conclusions 

The study focused on composites made of glass fibers. The key qualities of concrete, such as mechanical durability and microstructure, were considered. Following are the conclusions reached after a thorough examination and discussion of the findings.

- Glass fibers have a negative impact on concrete flowability due to their higher surface area, which increases resistance to flow.
- Glass fibers did not show significant improvement in compressive strength. Tensile and flexural capacity, on the other hand, were significantly enhanced. This is a result of the glass fibers’ ability to resist cracking.
- The surface hardness of glass fiber-reinforced concrete is lower than that of steel fiber-reinforced concrete, which is rated as a good layer.
- Glass fibers reduce the permeability of concrete to chloride-ions due to crack prevention.
- Glass fibers increase the performance of concrete in a maritime environment.
- The optimal dosage of fibers is affected by the water to binder ratio, according to preliminary study of SEM observation. The major causes for this are the dramatic increases in pore size and quantity caused by the coupling effect of increased water/binder ratio and fiber content. The impact of the water/binder ratio should be addressed when considering the effect of fibers on the mechanical or microstructural qualities of concrete.

## 9. Recommendations for Future Study

- The performance of concrete in harsh environments should be thoroughly examined.
- With regard to the creep and dry shrinkage qualities of glass fiber-reinforced concrete, there is no information available.
- It is essential to explore the thermal characteristics of glass-based composites.
- According to some research, glass fibers do not considerably improve the compressive capacity of concrete. As a result, further research using various pozzolanic materials is needed to increase the compressive capacity of glass fiber-reinforced concrete.

## Figures and Tables

**Table 1:** Physical properties Glass Fibers (GF).

Reference	[30]	[31]	[32]	[24]	[33]
Specific gravity	-	-	2.60	-	2.7
Water Absorption (%)	-	-	-	-	-
Stiffness (KN/mm)	72	-	-	-	-
Fiber length (mm)	-	12	6–18	12	-
Density (g/cm^3^)	2.58	-	-	2.60	-
Tensile strength (MPa)	2200	3400	1000–1700	3400	-
Tensile modulus (GPa)	70	-	-	-	-
Modulus of elasticity (GPa)	29	77	72	77	80.4

**Table 2:** Summary of Fresh and Mechanical performance of concrete with Glass Fibers (GF).

Reference	Glass Fibers	Slump (mm)	Compression Strength (MPa)	Split Tensile Strength (Mpa)	Flexure Strength (Mpa)
[32]	For NCA	-			
	0%		38.94	3.14	3.58
	0.25%		42.71	3.57	4.59
	0.50%		42.26	3.71	4.47
	0.75%		41.46	3.65	4.39
	For RCA				
	0%		34.26	2.79	3.29
	0.25%		36.24	3.29	4.42
	0.50%		37.32	3.42	4.34
	0.75%		35.98	3.35	4.21
[65]	GF BA	-	7 days 28 days	28 days	28 days
	0% 10%		32.00 47.50	3.19	6.55
	0.50% 10 %		36.60 52.00	3.35	6.83
	1.00% 10%		36.65 53.12	3.43	6.93
	1.50% 10%		37.23 54.29	3.51	7.05
	2.00% 10%		36.70 53.70	3.4	6.98
[71]		-	7 days 28 days	28 days	28 days
	0%		28.6 28.6	5.46	7.18
	0.45%		32.5 25.5	4.75	7.69
	0.90%		32.0 42.2	6.26	9.2
	1.35%		34.3 43.9	7	10.01
[56]	0%	-	40	-	-
	0.25%		52.21		4.88
	0.75%		55.83		5.36
	1.25%		59.17		5.98
[24]	0%	18	63	3	5
	0.25%	12	62	3.5	6.5
	0.50%	8	62	3.7	6.5
	0.75%	11	67	3.8	6
	1.00%	8	64	3.4	6.3
[72]		-	WC OC	WC OC	-
	0%		61.45 54.8	3.85 3.23	
	5%		70.25 66.17	4.18 3.44	
	15%		65.21 59.77	4.22 4.19	
[73]		-	14 days 28 days	14 days 28 days	14 days 28 days
	0%		31.36 39.85	2.13 2.83	2.94 3.02
	0.50%		26.88 34.07	2.59 2.68	2.89 3.76
	1.00%		36.88 41.63	2.60 3.48	5.42 6.63
	1.50%		30.37 36.00	2.78 2.82	6.35 6.48
[49]			7 days 28 days	-	7 days 28 days
	2.00%	162	35 45		8.0 12.0
	2.50%	157	36 46		8.5 12.5
	3.00%	152	37 47		8.7 12.7
	3.50%	149	36 46		8.0 13.0
	GF SF	-	-		
	100% 0%			181.84 179.29	288.80 272.52
	90% 10%			168.82 173.96	269.32 258.58
[30]	80% 20%			171.25 185.25	212.32 232.65
	70% 30%			158.69 135.23	234.12 217.38
	60% 40%			142.32 138.28	208.45 205.15
[70]	0%	-	72	5.7	5.9
	0.10%		70	5.8	6.1
	0.20%		69	6.1	6.1
	0.30%		68	6.1	6.3
	0.40%		67	6.2	6.4
	0.50%		66	6.2	6.6
	0.60%		65	6.5	6.6
	0.70%		52	6.5	6.7
	0.80%		48	6.5	6.7
[74]	0%	-	34		3.6
	0.25%		35		4.2
	0.50%		37		4.9
	1.00%		35		4.8
[31]			28 days 90 days	28 days 90 days	28 days
	0%		72.0 86	3.10 3.60	5.7
	0.25%		71.0 86	3.15 3.60	-
	0.50%		70.5 87	3.20 3.70	5.7
	0.75%		70.5 88	3.40 3.90	5.6
	1.00%		73.5 92	3.60 4.10	5.7
	1.25%		74.0 93	3.80 4.20	-
	1.50%		74.5 95	3.90 4.25	6.5
[75]		-	-	-	28 days 56 days
	0%				6.8 7.5
	5%				8.0 9.0
	6%				8.5 9.0
	7%				8.0 9.0
[76]			VC SC	VC SC	VC SC
	0%	55	47.77 54.44	3.60 3.85	4.36 4.74
	0.02%	48	50.19 57.55	3.78 4.13	4.56 5.05
	0.03%	45	53.66 61.33	3.92 4.24	4.66 5.17
	0.04%	42	57.33 65.55	4.10 4.39	5.13 5.61
[45]	0%	205	64.4	-	6.8
	0.50%	185	66		7.6
	0.75%	170	60		8
	1.00%	155	54		7.4
	1.25%	140	70		9.1

Bagasse Ash = (BA), Water curing = WC, Oven Curing = OC, Glass fiber = GF, Sisal fiber = SF, Vibrated concrete = VC. Self-compacting concrete = SC.

## Data Availability

All the data are available in manuscript.
